# Interactions of Colorectal Cancer, Dietary Fats, and Polymorphisms of Arachidonate Lipoxygenase and Cyclooxygenase Genes: A Literature Review

**DOI:** 10.3389/fonc.2022.865208

**Published:** 2022-07-19

**Authors:** Maryam Gholamalizadeh, Nazanin Majidi, Shirin Tajaddod, Sepideh Abdollahi, Seyed Mohammad Poorhosseini, Mina Ahmadzadeh, Mohammad Naimi Joubani, Samaneh Mirzaei Dahka, Hanieh Shafaei, Mogge Hajiesmaeil, Atiyeh Alizadeh, Saeid Doaei, Anahita Houshiar-Rad

**Affiliations:** ^1^ Cancer Research Center, Shahid Beheshti University of Medical Sciences, Tehran, Iran; ^2^ Department of Nutrition, Science and Research Branch, Islamic Azad University, Tehran, Iran; ^3^ Department of Medical Genetics, School of Medicine, Tehran University of Medical Sciences, Tehran, Iran; ^4^ Department of Medical Genetics, Faculty of Medicine, Shahid Beheshti University of Medical Sciences, Tehran, Iran; ^5^ Department of Clinical Nutrition and Dietetics, Faculty of Nutrition and Food Technology, National Nutrition and Food Technology Research Institute, Shahid Beheshti University of Medical Sciences, Tehran, Iran; ^6^ Research Center of Health and Enviroment, School of Health, Guilan University of Medical Sciences, Rasht, Iran; ^7^ Nursing and Midwifery School, Guilan University of Medical Sciences, Rasht, Iran; ^8^ Department of Biology and Biotechnology ”Charles Darwin”, Sapienza University of Rome, Rome, Italy; ^9^ Department of Pharmacognosy, Faculty of Pharmacy, Tehran University of Medical Sciences, Tehran, Iran; ^10^ Reproductive Health Research Center, Department of Obstetrics and Gynecology, School of Medicine, Al-Zahra Hospital, Guilan University of Medical Sciences, Rasht, Iran; ^11^ Dept. of Nutrition Research, Faculty of Nutrition Sciences and Food Technology, National Nutrition and Food Technology Research Institute, Shahid Beheshti University of Medical Sciences, Tehran, Iran

**Keywords:** colorectal cancer, polymorphism, dietary fat, lipoxygenase, cyclooxygenase

## Abstract

**Objective:**

Genetics and dietary factors play important roles in the development of colorectal cancer (CRC). However, the underlying mechanisms of the interactions between CRC, gene polymorphisms, and dietary fat are unclear. This review study investigated the effects of polymorphisms of arachidonate lipoxygenase (*ALOX*) and cyclooxygenase (*COX*) genes in the association between CRC and dietary fat.

**Methods:**

All the related papers published from 2000 to 2022 were collected from different databases such as PubMed, Science Direct, Scopus, and Cochran using related keywords such as colorectal cancer, *ALOX*, *COX*, polymorphism, and dietary fat. Non-English and unrelated documents were excluded.

**Results:**

Some single-nucleotide polymorphisms (*SNPs*) in the *ALOX* and *COX* genes, such as rs2228065, rs6413416, and rs4986832 in the *ALOX* gene, and rs689465 in the *COX* gene may play significant roles in the association between the risk of CRC and dietary fats. SNPs of ALOX and COX genes may influence the effects of dietary fatty acids on the risk of CRC.

**Conclusion:**

Some polymorphisms of the *ALOX* and *COX* genes may have important roles in the effects of dietary fat on the risk of CRC. If future studies confirm these results, dietary recommendations for preventing colorectal cancer may be personalized based on the genotype of the *ALOX* and *COX* genes.

## Introduction

Colorectal cancer (CRC) is the second leading cause of cancer death in women and the third in men worldwide (1) and cause about 0.9 million deaths worldwide in 2020 (2). It has been reported that CRC originates from a combination of genetic, environmental, and behavioral risk factors. Some behavioral factors are associated with dietary intake, including higher intake of calories, red meat, and fats (3, 4).

Recently, various types of fatty acids have been reported as effective dietary factors in CRC development. Some fatty acids, such as saturated fatty acids, may have an adverse effect, whereas other fatty acids, such as omega-3 fatty acids, may have a beneficial effect on CRC prevention (5–7). One main mechanism through which dietary polyunsaturated fatty acids (*PUFAs*) may affect colonic carcinogenesis is the formation of specific eicosanoids (oxygenated metabolites of *PUFAs*) such as prostaglandins (*PGs*), thromboxanes (*TXs*), leukotrienes (*LTs*), and lipoxins (*LXs*) (7). Two enzymatic pathways related to the synthesis of these eicosanoids are the arachidonic lipoxygenase (*ALOX*) pathways and prostaglandin-endoperoxide synthase (*PTGS*), which are also known as cyclooxygenase (*COX*) pathways (8). The function of ALOX enzymes, such as ALOX5, ALOX12, and ALOX15, eventually leads to LT and LX formation, and the COX enzymes, like COX1 and COX2, result in the production of PGs and TXs (9, 10). Evidence has shown that changes in the sequence of *COX* and *ALOX* genes as single-nucleotide polymorphisms (*SNPs*) can influence the risk of CRC (8).

In terms of *ALOX*, previous research has established that *ALOX*15 expression and concentration of eicosanoic metabolites are reduced in polyps and colorectal tumors in humans (11). In contrast, increased *ALOX*5 expression has been reported in colorectal cancer cells (12). Moreover, it has been reported that some mutations in *ALOX*12 are associated with tumorigenesis in epithelial cancers (13). Recent studies have identified that the expression level of the *COX2* gene and the levels of its metabolites, such as *PGE_2_*, *PGD_2_*, and *PGF2α*, are significantly increased in the colon of obese mice. Also, it has been shown that the administration of *COX2* inhibitors can suppress inflammation, tumor growth, and tumor metastasis (14–16).

Notably, the effect of dietary fats on the risk of CRC may be influenced by gene polymorphisms (17–19). However, the interactions between CRC, dietary fat, and gene polymorphisms are still unknown. So, this review study investigated the effects of SNPs of the *ALOX* and *COX* genes on the association between dietary fats and CRC risk.

## Materials and Methods

### Search Strategy

The literature search was performed using the PubMed, Science Direct, Scopus, and Cochran databases, and all related papers published from 2000 to 2022 were collected using the following keywords: “dietary fat or fatty acid or fat or lipid” and “*ALOX* or *COX* or prostaglandin-endoperoxide synthase or *PTGS* or cyclo-oxygenase or *COX* or arachidonic lipoxygenase or lipoxygenase” and “colorectal cancer or colon cancer or rectal cancer” and “polymorphism or genetic variation or genotype or *SNP.*” All the collected papers and their references were reviewed.

### Inclusion and Exclusion Criteria

All studies that examined the interaction of colorectal cancer with *ALOX* and *COX* genes, studies concerning the relationship between the *ALOX* and *COX* gene polymorphisms, and studies on the interactions between colorectal cancer, *ALOX* and *COX* genes, and dietary fat were included in this study. Unrelated and non-English papers, the review studies, studies on the relationship between *ALOX* and *COX* with other cancers, and animal studies were excluded.

### Assessment of Methodological Rigor

In this review study, the quality of the collected studies was assessed by four researchers (MG, HS, SA, and SD). In the case of having opposing ideas, other researchers (MH and HS) would be involved in reaching an agreement. After collecting the papers, all unrelated studies were excluded from the review process according to their titles and abstracts. Then, the full texts of the relevant articles were studied precisely. The standard quality assessment method of the ‘EPOC Risk of Bias Tool’ was applied to assess the quality of the methodologies (20). The Preferred Reporting Items for Systematic Reviews and Meta-Analyses (*PRISMA*) checklist (21) was used to extract the required data from the included studies. Finally, the data about the participants, intended comparisons, obtained results, and study planning (*PICOS*) were collected.

## Results

### Description of the Identified Studies

**Figure 1:**
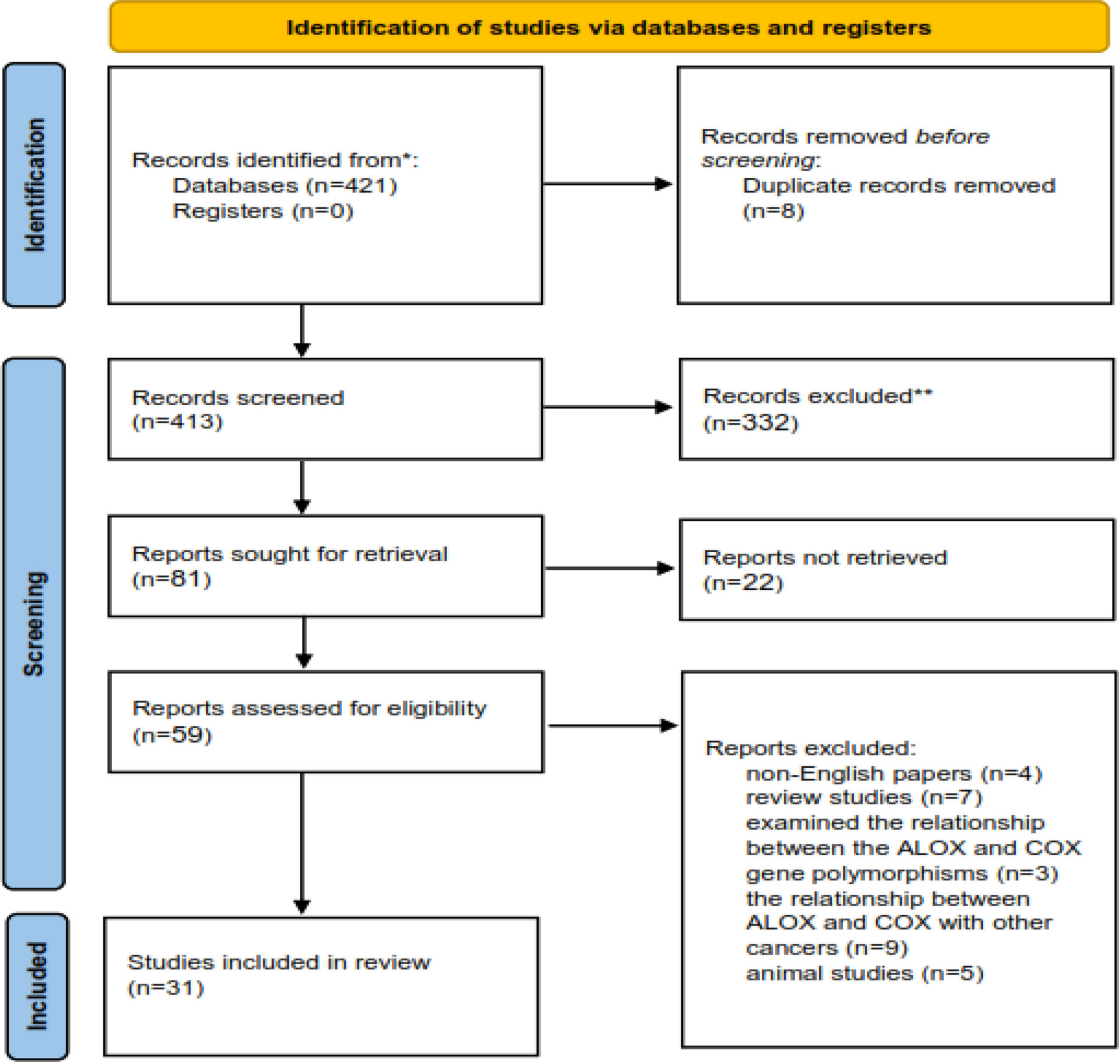
The process of including the appropriate studies.

The process of including the appropriate studies is presented in **Figure 1**. A total of 413 articles were collected in the primary search, of which 354 articles were excluded after the screening of their titles and abstracts. Also, 28 articles were excluded after reading the full texts. Finally, 31 articles qualified to be included in the review process. All articles were published from 2000 to 2022 and were related to the interactions between *CRC, ALOX*, and *COX* genes and fat intake. The main characteristics of the studies are presented in **Tables 1**, **2**.

**Table 1:** Summary of the studies related to *ALOX* gene polymorphisms and CRC risk.

Study	Ethnicity	Study design	Case/Control	Polymorphisms	Main finding
Goodman et al. (8)	African-Americans and Caucasians	Case–control study	468 cases and 304 controls	rs6413416, rs4986832 and rs2228065 in *ALOX*5, and rs1126667 in *ALOX*12	This study found that a haplotype including *ALOX*5 rs6413416 and rs4986832 was associated with decreased colorectal cancer risk in Caucasians.
Kleinstein et al. (22)	American	Case–control study	Colon cancer (1,424 cases/1,780 controls) rectal cancer (583 cases/775 controls), colorectal adenomas (485 cases/578 controls)	Four SNPs in FLAP (rs17239025), *ALOX* 12 (rs2073438), and *ALOX*15 (rs4796535 and rs2619112)	*ALOX12* (rs2073438) was associated with a lower risk of rectal cancer. *ALOX15* (rs4796535 and rs2619112) was associated with an increased risk of rectal cancer.
Tan et al. (23)	Chinese	Case–control study	1,000 cases and 1,300 controls	*ALOX12* (rs1126667)	*ALOX 12* rs1126667 was associated with a moderately increased risk of CRC.
Poole et al. (24)	Minnesota	Case–control study	517 adenomatous or 192 hyperplastic polyps versus 618 polyp-free controls	*ALOX*5 (rs4986832)	ALOX5 rs4986832 polymorphism did not have any association with the risk of colorectal polyps.
Ruan et al. (25)	China	Cross-sectional	438 tumor tissue samples and 41 adjacent tissue samples	*ALOX* gene family expression (*ALOX*E3, *ALOX*5, *ALOX*12, and *ALOX*12B)	The ALOX12 mRNA expression could be a diagnostic marker for colon adenocarcinoma and the expression of ALOXE3 combined with ALOX12 could have a prognostic value in colon adenocarcinoma.

ALOX, Arachidonic Acid Lipoxygenase; FLAP, Arachidonate 5-lipoxygenase-activating protein; CRC, Colorectal cancer.

**Table 2:** Summary of the studies related to *COX* gene polymorphisms and CRC risk.

Study	Ethnicity	Study design	Case/Control	Polymorphisms	Main finding
Lin et al. (26)	African-American, Chinese (Hong Kong), Filipino, Hispanic, Indian (Asian), Japanese, Korean, Samoan, and Caucasian.	Case–control study	299 cases and 477 controls	V511A (rs5273) in PTGS2(*COX*2)	The *COX*2 rs5273 polymorphism may reduce the risk of CRC in African-Americans
Cox et al. (27)	Chinese	Case–control study	292 cases and 272 controls	COX2 rs4648298, rs689469, rs689165, rs20417, rs20424, rs5277, rs20432, rs5275	COX2 rs4648298 and rs689469 polymorphisms had an association with an increased risk of CRC
Mosallaei et al. (28)	Isfahan, Iran	Case–control study	88 cases and 88 controls	COX2 rs4648298 polymorphism	There was a significant relationship between AA genotype and CRC risk reduction in the Iranian population (OR=0.14; 95% CI, 0.05-0.34; P <0.001).
Ulrich et al. (29)	American	Case–control study	680 cases and 584 controls	*COX*2 (rs20417)	The allele frequencies of *COX*2rs20417reduced the risk of CRConly among non-users of NSAIDs.
Hoff et al. (30)	Caucasian	Case–control study	326 cases and 369 controls	The *COX*2 rs20417 and rs689466	The -765GG genotype (rs20417) increased CRC risk, while GG/AC haplotype (rs20417) decreased CRC
Xing et al. (31)	Asian	Case–control study	137 cases and 199 controls	*COX*2 rs20417	COX2 rs20417 polymorphism appears to be related to an increased risk of CRC in the smoker.
Ueda et al. (32)	Winston-Salem and Charlotte, North Carolina	Case–control study	162 incident, sporadic colorectal adenoma cases and 211 controls	COX2 (765G>C, 8473T<C, 9850 A>G),COX1 (842 A<G)	COX2 8473T>C can reduce the CRC risk in individuals who consume NSAIDs drugs
Shomaf et al. (33)	Caucasian	Case–control study	239 cases and 115 controls	*COX*2 rs689466	COX2 rs689466 polymorphism may have a protective role against the risk of CRC.
Peters et al. (34)	Caucasian	Case–control study	85 cases and 218 controls	*COX*2 rs689466	There was overexpression of COX2 rs689466 GG genotype compared with AA genotype in patients with FAP.
Pereira et al. (35)	Caucasian	Case–control study	246 cases and 480 controls	*COX*2 rs689466	There was a nearly 6-fold increased CRC risk in smoker individuals with COX2 rs689466.

PTGS2, Prostaglandin-Endoperoxide Synthase 2; COX2, Cyclooxygenase; CRC, Colorectal cancer; NSAIDs, Non-steroidal anti-inflammatory drugs; FAP, Familial adenomatous polyposis.

### Arachidonate Lipoxygenase (*ALOX*) Gene Polymorphisms and Risk of CRC

**Figure 2:**
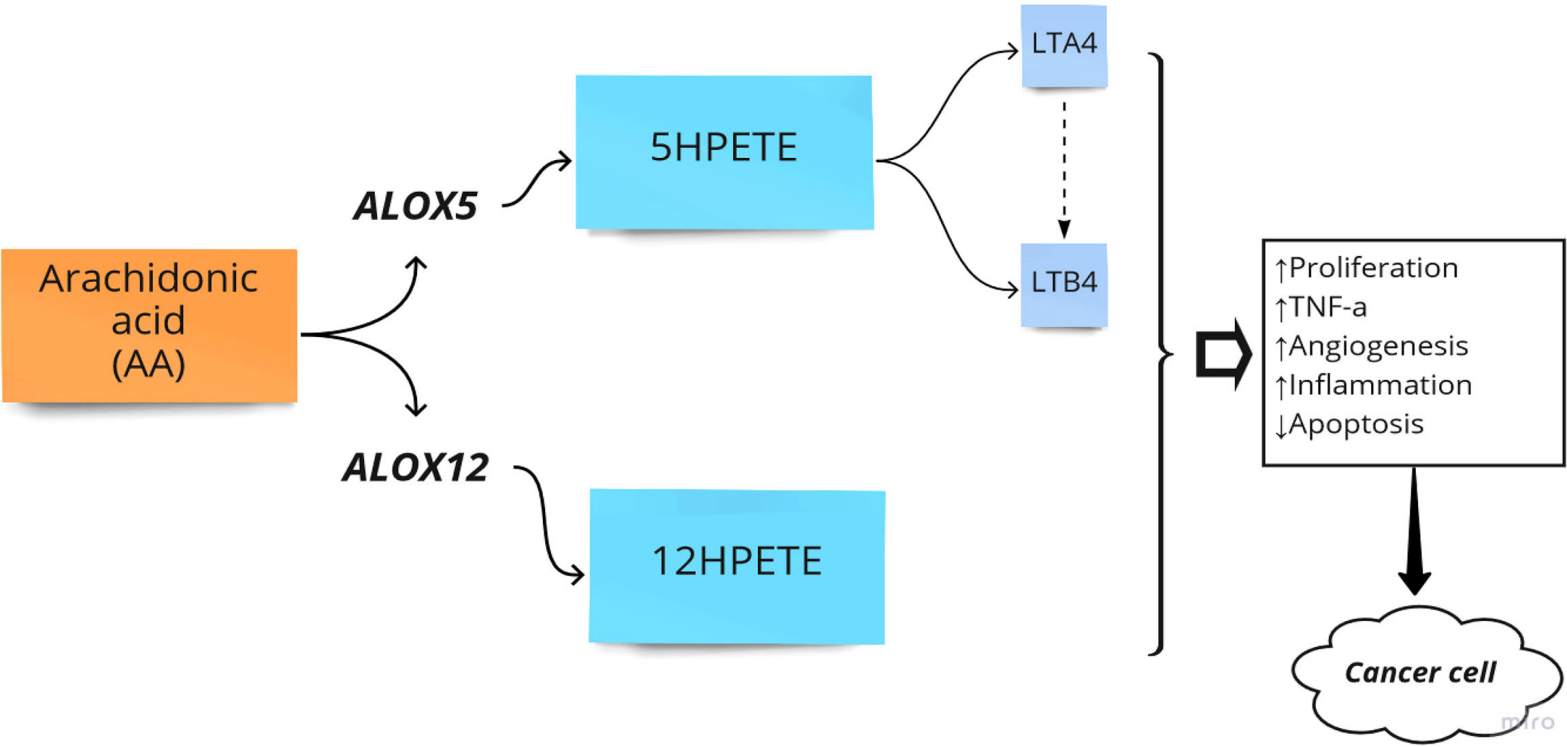
Arachidonate lipoxygenase (ALOX) in the metabolism of Arachidonic Acid (AA). HPETE, hydroperoxyeicosatetraenoic acid; LT, Leukotriene; ↑, Increase; ↓, Decrease.

The primary function of *ALOX* is to convert arachidonic acid (*AA*) into hydroperoxyeicosatetraenoic acid (*HPETE*) and eventually leukotrienes, a class of paracrine hormones involved in the inflammatory response. For example, *ALOX*12 converts AA into 12-hydroperoxyeicosatetraenoic acid (*12-HPETE*), which is involved in the expression of pro-inflammatory cytokine genes such as tumor necrosis factor-α (*TNF-α*) (36). The role of the *ALOX* gene in inflammatory diseases and colorectal neoplasia has been frequently reported. For example, the arachidonate-5 lipoxygenase (*ALOX*5) and 12-lipoxygenase (*ALOX*12) played pro-carcinogenic roles in colorectal cancer (22). Additionally, overexpression of *ALOX*5 with its related downstream metabolites has been reported in other cancers such as breast, esophageal, pancreatic, and prostate cancers by stimulation of cell proliferation, tumor angiogenesis, and survival (37). Moreover, it has been reported that *ALOX*15 is associated with an increased risk of colorectal cancer, particularly in people with higher inflammatory factors (38). In another study, Ruan et al. examined the diagnostic and prognostic values of the *ALOX* gene family mRNA expression in 438 colon adenocarcinoma tumor samples and 41 adjacent tissue samples of Chinese patients by bioinformatics analysis. They showed that the expression level of *ALOX*E3, *ALOX*5, *ALOX*12, and *ALOX*12B was upregulated in colorectal tumor samples. Finally, they reported that *ALOX*E3 and *ALOX*12 might serve as potential independent prognostic indicators of colon adenocarcinoma (25). Thus, *ALOX* pathways in the *AA* metabolism process can be considered crucial pathways in the development of CRC (**Figure 2**).

Notably, specific polymorphisms of the *ALOX* gene can affect the susceptibility to CRC. For instance, Goodman et al. assessed the effects of *ALOX*5 and *ALOX*12 gene polymorphisms on CRC in African-Americans and Caucasian patients. They found that the rs6413416 and rs4986832 polymorphisms of *ALOX*5 were associated with a decreased risk of CRC in Caucasians. They hypothesized that these polymorphisms could improve binding to the promoter region, leading to downregulation of *ALOX*5. In this way, they can lower the cancer risk by reducing enzymatic activity (8). However, the rs4986832 polymorphisms of *ALOX*5 had no association with the risk of colorectal polyps in Minnesota (24). Kleinstein et al. conducted a study on 2447 cases and 3133 controls regarding the effect of *ALOX* gene polymorphisms on the risk of CRC. The results showed that the rs2073438 polymorphism of *ALOX* 12 was related to a lower risk of rectal cancer (OR = 0.66, 95% CI: 0.42–1.04), while the rs4796535 and rs2619112 polymorphisms of *ALOX*15 were associated with an increased risk of rectal cancer (OR = 1.43, 95% CI: 1.03–1.97 and OR = 1.13, 95% CI: 0.85–1.55, respectively) (22). Moreover, a positive association was found between the *ALOX*12 rs1126667 polymorphism and a moderately increased risk of CRC (OR = 1.38, 95% CI: 1.09–1.74) (23). However, the association between rs1126667 polymorphism of *ALOX*12 and the risk of CRC has been reported in African-Americans and Caucasian patients (8). This discrepancy can be due to differences in ethnic or statistical power. A summary of studies on the association between *ALOX* polymorphisms and CRC is provided in **Table 1**.

### Cyclooxygenase (*COX*) Gene Polymorphism and Risk of CRC

**Figure 3:**
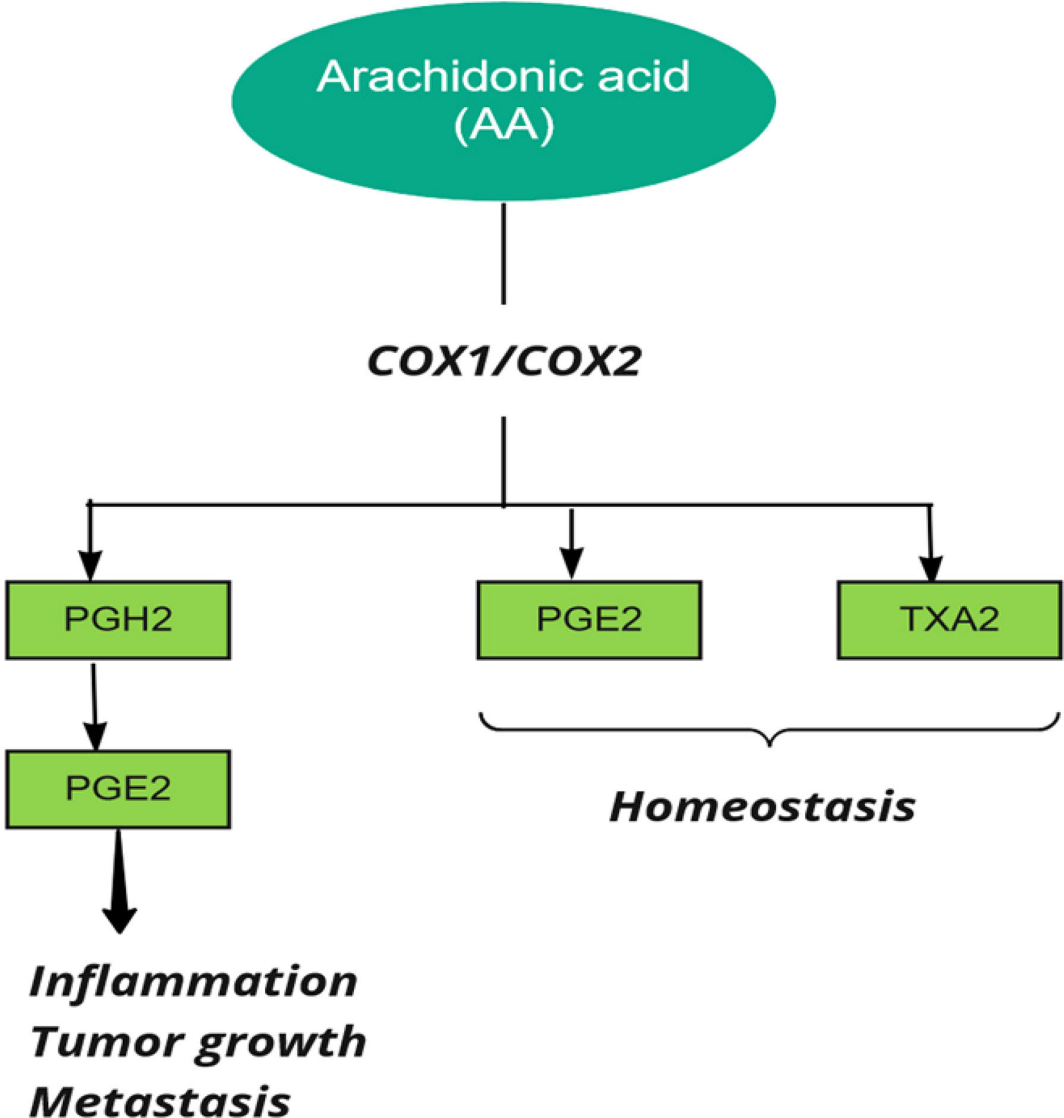
Cyclooxygenase (COX) in metabolism of Arachidonic Acid (AA). PG, Prostaglandin, TX, Thromboxane.

Prostaglandin H synthase, also known as cyclooxygenase and prostaglandin-endoperoxide synthase (*PTGS*), catalyzes the first step in the biosynthesis of all prostaglandins and prostacyclins by converting arachidonic acid to prostaglandin H (39). Two forms of human *PTGS*, *PTGS1* and *PTGS2* (*COX1* and *COX2*), can be inhibited by non-steroidal anti-inflammatory drugs (*NSAIDs*). Also, the end products of *COX* are related to various biological pathways in stimulating tumor growth (26) (**Figure 3**).

Prostaglandins are upregulated in colorectal cancer, and it was reported that genetic polymorphisms in both *COX1* and *COX2* are associated with CRC (27, 37). *COX2* is involved in cell cycle control, and increased expression of *COX-2* in CRC patients compared to normal controls indicates its possible role in the progression of CRC (40). *COX2* influences cancer progression by increasing prostaglandin production, preventing tumor cell apoptosis, cell proliferation, and tumor angiogenesis (41). It was reported that aspirin plays a key role in preventing colon cancer by inhibiting *COX* (37). Ayiomamitis et al. examined the expression of *COX1*, *COX2*, prostaglandin-endoperoxide synthase 3 (*PTGES3*), and telomerase reverse transcriptase (*TERT*). They used bioinformatics analysis on the Cancer Genome Atlas Colon Adenocarcinoma (*TCGA*-*COAD*) and rectal adenocarcinoma (*READ*) datasets. The results showed an inverse relationship between *COX2* expression and telomerase activity in CRC. In the end, they identified differentially methylated patterns within the promoter regions of *COX2* and *TERT* (42). Joanna et al. observed *COX2* overexpression in the early stages of colorectal cancer and higher *COX2* gene expression in the advanced stages of the disease. The results also indicated that *COX2* expression level could affect carcinogenicity by modulating local inflammation (43). In addition, a case–control study on Iraqi patients reported that the expression level of *COX2* was upregulated at higher tumor grades (44). This result suggests that considering *COX2* as an early marker of progression or initiation of colorectal carcinoma should be investigated by further studies. Moreover, Labda et al. found that *COX2* expression was associated with tumor size and degree of differentiation in an observational study including 58 Indonesian CRC patients. However, there was no statistical correlation between *COX2* expression and tumor location (45). Jin et al. conducted a case-control study involving 213 Chinese patients with colorectal cancer and 200 controls and reported that the expression level of *IGF-IR* and *COX2* was directly related to the degree of progression and lymphatic metastasis and inversely related to the mean survival rate in CRC patients (46). The results of a Chinese study also indicated that *PGE_2_* and *COX2* expression were significantly associated with tumor invasion, tumor differentiation, lymph node metastasis, and *TNM* stage and were inversely related to patient survival (47).

Polymorphisms of the *COX* gene can affect the risk of *CRC*. In this regard, Lin et al. found that the *COX2* rs5273 polymorphism, in about 5% of African Americans, was associated with a lowered risk of CRC (OR = 0.78, 95% CI: 0.49–1.23) (26). The *COX2* rs4648298 and rs689469 polymorphisms were reported to be associated with an increased risk of CRC. Analysis of haplotypes confirmed that people with these variants were at an increased risk of colorectal cancer (OR = 2.17, 95% CI: 0.97–4.84, P = 0.06) (27). In contrast with these results, Mosallaei et al. observed a significant relationship between *COX2* rs4648298 polymorphism (*AA* genotype) and a reduced risk of CRC in the Iranian population (OR = 0.14; 95% CI: 0.05–0.34; P <0.001). Interestingly, they found this significant association only in non-smokers (28). This finding suggests that environmental factors may influence the association between the *COX* gene polymorphism and CRC. Another study showed that the *GG* genotype of *COX2* rs20417 was associated with an increased risk of developing CRC in the Dutch population (OR, 1.45; 95% CI, 1.03–2.04) (30). Interestingly, Xing et al. observed the positive association between the *GG* genotype of the *COX2* rs20417 polymorphism and increased CRC risk in China, especially in smokers and in people with a high Body Mass Index (*BMI*) (OR: 1.107, 95% CI: 1.107–3.726; P = 0.022) (31). In this line, the Minnesota-based case-control study discovered that *COX2* gene expression or COX*2* enzyme activity is suppressed and the risk of colorectal polyps is reduced by NSAIDs in individuals with the GG genotype of *COX2* rs20417 (OR: 0.66; 95% CI: 0.48–0.92) (29). Ueda et al. investigated the association between the *COX2* position 765 G<C, 8473 T>C, and 9850 A>G and CRC risk and reported that among the studied polymorphisms, *COX2* 8473T>C may reduce the CRC risk in people who consume *NSAIDs* (OR: 1.57, 95% CI: 1.04–2.38) (32). Another previous study on 104 cases of adenomatous polyps and 115 matched control samples found that *COX2* rs689466 polymorphism may have a protective effect on the risk of development of CRC (33). In contrast, Peters et al. identified that overrepresentation of *COX2* was associated with a high risk for CRC development in patients with familial adenomatous polyposis (*FAP*) who had the rs689466 polymorphism *GG* genotype compared with *AA* genotype carriers (OR = 2.81; 95% CI = 1.00–7.91, P= 0.042) (34). In addition, Pereira et al. suggested that smoker people with *COX2* rs689466 polymorphism had a nearly 6-fold increased CRC risk compared with people without rs689466 risk allele (95% CI: 1.49–22.42, P  =  0.011) (35). Some reasons for these conflicting results on the association between *CRC* and *COX* gene can be due to effects of different environmental factors such as lifestyle on this association. **Table 2** presents the summary of studies regarding *COX* polymorphisms and CRC risk.

### Interaction Between CRC, *ALOX* and *COX* Polymorphisms, and Dietary Fatty Acids

**Figure 4:**
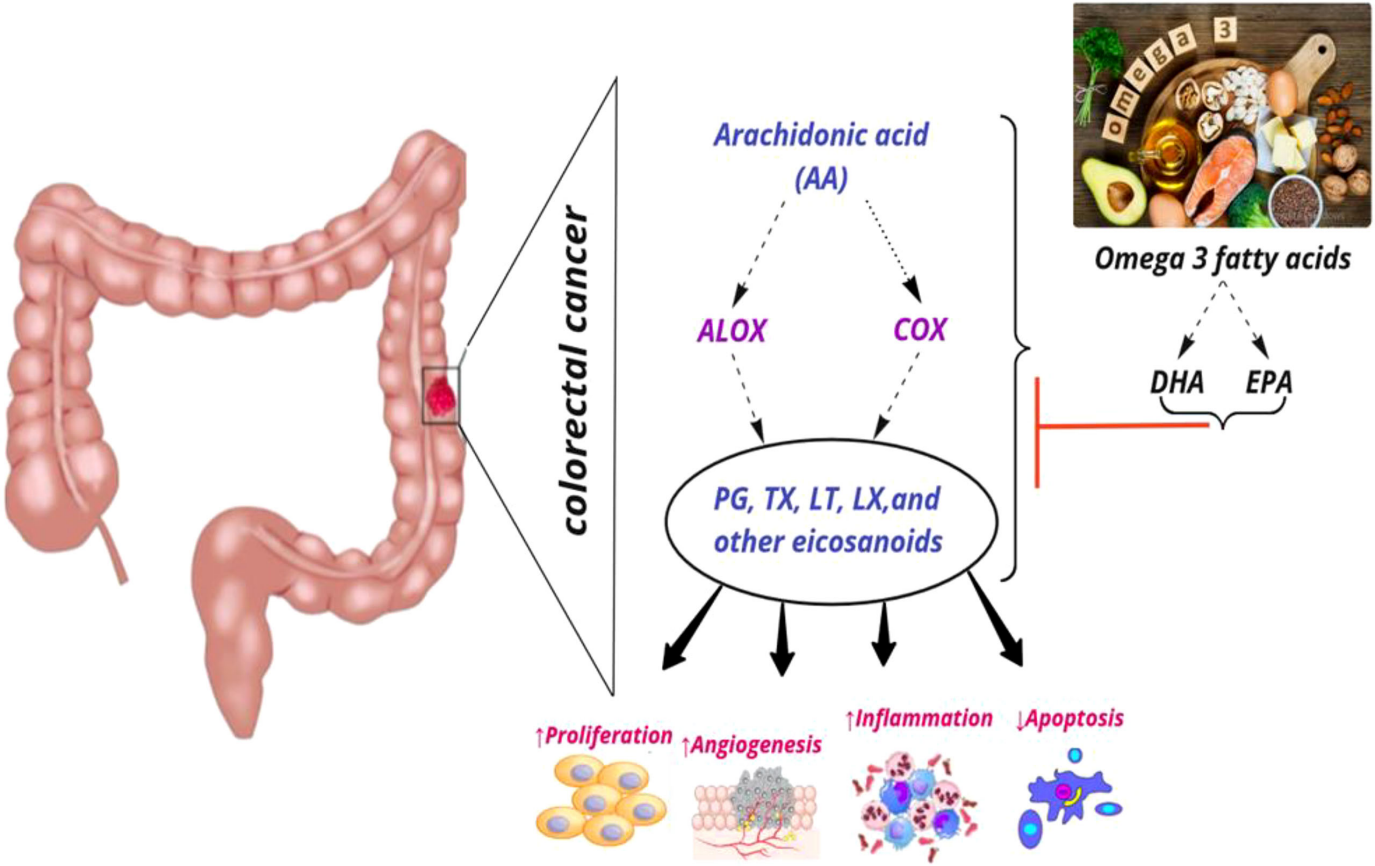
Interaction among dietary fatty acids, ALOX (Arachidonate lipoxygenase) and COX (Cyclooxygenase) in metabolic pathway of AA (Arachidonic Acid), and CRC (colorectal cancer) risk. PG, Prostaglandin; TX, Thromboxane; LT, Leukotriene; LX, Lipoxin; DHA, Docosahexaenoic acid; EPA, Eicosapentaenoic acid; ↑, increase; ↓, decrease.

*COX* enzymes (*COX1*, *COX2*) are important factors in the biosynthetic pathway of *PGs* from *AA*. *ALOX* enzymes (*ALOX5*, *ALOX12*, and *ALOX15*) convert *PUFA* to fatty acid hydroperoxides, which results in the production of *LTs*. Recent studies reported an association between the CRC risk with the amount of fatty acids intake and *COX* and *ALOX* polymorphisms (**Figure 4**). Habermann et al. identified an association between *COX1* rs10306110 polymorphism and low intake of docosahexaenoic acid (*DHA*), a fatty acid with anti-inflammatory properties, with an increased risk of colon cancer (OR = 1.6, 95% CI: 1.1–2.3, adjusted *P* = 0.06) (38). Notably, supplementation with some fatty acids such as ω-3 fatty acids plays a protective role in colon cancer by attenuating the pro-inflammatory state and decreasing the production of *PGE_2_* (48). These results conform to studies that indicated that omega-3 long-chain polyunsaturated fatty acids (n−3 *LC-PUFA*) may lower cancer risk by suppressing oxidative stress, tumor apoptosis, and inflammatory pathways by modulation of *COX* activity and inhibition of arachidonic acid-derived eicosanoids (49–51). However, a case–control study on 310 patients with colorectal cancer and 1,177 controls provided epidemiological evidence for the possible link between *PGs* production from n−6 *PUFAs* through the enzymatic activity of *COX2* and increased risk of colon cancer. They reported an association between *COX2* rs20417 polymorphism and an increased risk of colon cancer in individuals with high n−6 *PUFA* intake (OR = 2.38, 95% CI = 1.23–4.59, P = 0.07). However, there was no association between this polymorphism and the risk of rectal cancer regardless of the dietary n−6 *PUFA* intake levels (52). These results emphasize the importance of lifestyle modification in the carriers of the high-risk allele of the *COX* gene. Moreover, Siezen et al. demonstrated that colorectal adenoma risk could be modified by the interaction between polymorphisms in *AA* pathway genes and fish consumption. They showed that the *COX2* rs5277 polymorphism in people with high fish consumption played a protective role against CRC compared with people with low fish intake (53). In another work, Siezen et al. confirmed the inverse association between high fish consumption and CRC risk. However, they could not find any significant interaction between CRC and *SNPs* in the genes involved in the *AA* pathway (54). Interestingly, another study indicated that the effects of n−3 *PUFA* intake and *NSAID*s on CRC may differ in people with *COX1* polymorphisms. Among the wild-type homozygous individuals (*PP* genotype) with *COX1* rs3842787 polymorphism, high fish consumption and regular use of *NSAIDs* were associated with a decreased risk of CRC. In comparison, an inverse association was observed in individuals with at least one risk allele (PL, LL genotypes) in the *COX-1* rs3842787 polymorphism (55). Furthermore, dietary supplementation with n−3 *PUFA*, particularly *DHA* and *EPA*, was reported to have antineoplastic effects on CRC by modifying the epigenetic modification like *DNA* methylation (56). **Table 3** summarizes studies regarding the association between *ALOX*, *COX* polymorphism, dietary fatty acid, and CRC risk.

**Table 3:** Summary of studies regarding interactions between *ALOX, COX* polymorphism, dietary fatty acid, and CRC risk.

Study	Ethnicity	Study design	Case/Control	Polymorphisms	Main finding
Habermann et al. (38)	American	Case–control study	1,574 colon cancer and 791 rectal cancer and 2969 control	COX1 (rs10306110 and rs10306122), COX2 (rs4648276), ALOX15 (rs11568131)	There was a positive association between low intake of DHA and increased risk of colon cancer with COX1 rs10306110. There was a positive association between higher inflammatory score and increased risk of colon cancer with wild type ALOX15 rs11568131. There was an inverse association between low total fat intake and rectal cancer risk with COX1 rs10306122. There was an inverse association between low inflammatory score and rectal cancer risk with COX2 rs4648276.
Wilson et al. (48)	American	Cross-sectional study	90 participant	*PTGS*	Supplementation of some kind of fatty acids like ω-3 fatty acids can have a protective role in colon cancer by decreasing the production of PGE_2_.
Koh et al. (52)	Asian	Nested Case–control study	310 colorectal cancer cases and 1177 controls	*COX*2 rs20417	It was a statistically significant association between COX2 rs20417 polymorphism and CRC risk among high consumers of dietary n−6 PUFA.
Siezen et al. (53)	Netherlands	Case–control study	384 cases and 403 polyp-free controls	COX2 rs5277	COX2 rs5277 polymorphism in people with high consumption of fish had a protective role against CRC compared with people with low fish intake.
Siezen et al. (54)	Netherlands	Case–control study	508 cases and 772 controls	PTGS1 and PTGS2	Although there was a significant reduction in cancer risk for individuals with COX2 rs5277 in combination with high fish intake, no significant interaction was observed between the SNPs in genes involved in AA metabolism and fish intake.
Poole et al. (55)	Minneapolis	Case–control study	522 adenomas, 194 hyperplastic polyps and 626 polyp-free controls	COX -1 rs3842787	The results suggested that among individuals with the wild-type homozygous (PP) in COX1 rs3842787, increased fish consumption was associated with a slight reduction in the risk of adenoma, Whereas among people who had at least one different allele (LL, PL) in COX1 rs3842787, an inverse association was observed.
Sarabi et al. (56)	Shiraz, Iran	Cell culture	5 human CRC cell lines	Polyunsaturated fatty acids DNA methylation (DNMT)	PUFA significantly suppressed DNMT3a and DNMT3b expression in SW742 cells (p < 0.05) and PUFA treatment tends to coordinately suppress the expression of DNMTs in four CRC cells lines.

COX, Cyclooxygenase; ALOX, Arachidonic Acid Lipoxygenase; DHA, Docosahexaenoic acid; PTGS, Prostaglandin-Endoperoxide Synthase; CRC, Colorectal cancer; PUFA, Polyunsaturated fatty acids; DNMT, DNA methyltransferases.

Regarding the interactions between *ALOX* and *COX* gene polymorphisms, Siezen et al. reported that these SNPs are associated with colorectal adenoma risk and that these associations are modified by fish consumption. No association was found between *SNP* rs5277 in the *COX2* gene and rs743646 in the *ALOX15* gene (53).

## Discussion

The results of this study indicated that some *SNPs* of the *ALOX* and *COX* genes can be associated with the interaction between dietary fats and the risk of CRC. The metabolizing effects of *ALOX* and *COX* enzymes on *AA* were reported to be associated with the production of carcinogenic factors in the colon (57). The association between *ALOX12* and colorectal neoplasia has been reported (22). However, Goodman et al. found that the *ALOX5* gene haplotype, including the rs6413416 and rs4986832 polymorphisms, was associated with a reduced risk of CRC in Caucasians. They assumed that these polymorphisms could augment the binding to the regulatory region of the promoter. Thus, attenuating the enzymatic activity could lead to a lower cancer risk. While this association was not observed in the African-American population, this inconsistency can be related to the existence of effective genetic or environmental factors in the African-American population (8).

Concerning the role of the *COX* enzyme in CRC risk, it has been reported that *COX2* is involved in the early stages of colon cancer development (42). Low *COX2* expression is observed in the early stages of colon cancer and *COX2* overexpression is more common in the advanced stages of the disease (43). *COX2* expression was significantly associated with CRC tumor invasion, tumor location, tumor size, degree of differentiation, and metastasis (45, 58). However, there was no significant relationship between *COX2* expression and the histological type of CRC (45). On the other hand, an inverse association was reported between CRC with *COX2* expression as well as methylation patterns within the promoter regions of *COX2* (42). Regarding the association between CRC and *COX*2 genotype, some studies found no association between rs20420 and rs5273 polymorphisms of the *COX2* gene and CRC risk (8, 59). In contrast, Lin and Schumaf reported the protective effect of *COX2* rs689466 and rs5273 polymorphisms against colorectal neoplasms (26, 33), and some other studies reported an increased risk of CRC carriers of some *COX2* polymorphisms such as rs689466 and rs20417 (31, 32, 35, 43). These conflicting results of the studies can be due to differences in ethnicity, environmental factors, and tumor type.

The role of polyunsaturated fatty acids (*PUFAs*) in the prevention of various types of malignancy, such as CRC, has been frequently reported (56). Recent studies found that dietary fatty acids may influence the association between CRC with ALOX and COX genes. For example, Habermann et al. indicated the effects of different fatty acid intake patterns on the association between colon cancer risk and *COX1* rs10306110 and *ALOX15* rs11568131 polymorphisms and also on the association between rectal cancer risk and *COX1* rs10306122 and *ALOX12* rs11571339 polymorphisms. They reported a possible increase in CRC risk among those with low intake of the marine sources of n−3 *PUFAs* such as *EPA* and *DHA* in people with a risk allele of *COX1* rs10306110 polymorphism (38). The evidence indicates that n−3 *LC-PUFA* may decrease cancer risk by suppressing oxidative stress, tumor apoptosis, and inflammatory pathways. They can decrease inflammation *via* the modulation of *COX* activity and inhibition of arachidonic acid-derived eicosanoids (49–51). It has also been reported that consuming higher n−3 fatty acids may reduce the production of pro-inflammatory eicosanoids, which may be involved in colon cancer (17). In this regard, Wilson et al. reported that ω-3 fatty acid supplementation upregulated *COX1* expression and reduced the pro-inflammatory state. Individuals with higher mRNA expression of *COX2* after ω-3 fatty acid supplementation had reduced colonic *PGE_2_* (48). The *ALOX* and *COX* gene expression level in CRC patients is supposed to be dependent on dietary fats. Koh et al. provided epidemiological evidence for a possible association between the production of prostaglandin n−6 *PUFAs* through *COX2* enzymatic activity and an increased risk of colon cancer. They also showed a significant association between the *COX2* rs20417 and colorectal cancer risk among people with a higher intake of n−6 *PUFA* (52). The ratio of omega-6 fatty acids (as precursors of inflammatory eicosanoids) to omega-3 fatty acids (as precursors of anti-inflammatory eicosanoids) may affect the extent to which the ALOX and COX genes affect colorectal cancer risk. These results highlight the importance of the intake of different types of dietary fats in carriers of the risk alleles of the ALOX and *COX* genes. However, few studies directly assess this interaction. Also, different factors, such as ethnic and racial differences, may influence the obtained results on the interactions of *CRC* risk with dietary fats and *ALOX* and *COX* genes. Further studies are needed to understand the interaction between dietary fat, genetics, and colorectal cancer. Moreover, other genes involved in enzymatic pathways for synthesizing eicosanoids from dietary fats and possible mechanisms for their relationship with CRC risk should be investigated. If the findings of this review study are confirmed in future longitudinal studies, it could be an important step in providing a specific diet to prevent colorectal cancer, especially in COX and ALOX risk allele carriers.

## Conclusion

In conclusion, COX and ALOX genes may play a significant role in CRC risk. Additionally, dietary fats may play an essential role in the effects of the *ALOX* and *COX* genes on the risk of CRC. Generally, enhancing the knowledge of nutritional genomics can lead to finding new methods to prevent, treat, and manage CRC. The results of this review article emphasize that environmental factors, such as dietary fat intake, may influence the association between colorectal cancer and the genotype of an individual. If the results are confirmed in future longitudinal studies, the importance of personalized medicine and the recommendation of personalized diets according to the genotype of individuals to prevent colorectal cancer will be further highlighted. Further longitudinal studies in this field of nutritional genomics can lead to the discovery of personalized dietary recommendations for CRC prevention.

## Author Contributions

MG, SMD, and AH designed the study, and were involved in the data collection, analysis, and drafting of the manuscript. NM, SA, SA, SP, MNJ, SD, HS, MH, MA, and AA were involved in the design of the study, analysis of the data, and critically reviewed the manuscript. All authors listed have made a substantial, direct, and intellectual contribution to the work and approved it for publication.

## Funding

Funding for this study was provided by School of Nutrition and Food Sciences, National Nutrition and Food Technology Research Institute, Shahid Beheshti University of Medical Sciences, Tehran, Iran (Code 27846).

## Conflict of Interest

The authors declare that the research was conducted in the absence of any commercial or financial relationships that could be construed as a potential conflict of interest.

## Publisher’s Note

All claims expressed in this article are solely those of the authors and do not necessarily represent those of their affiliated organizations, or those of the publisher, the editors and the reviewers. Any product that may be evaluated in this article, or claim that may be made by its manufacturer, is not guaranteed or endorsed by the publisher.
